# Transjejunal endoscopic ultrasound‐guided pancreatic drainage for pancreatic jejunostomy stricture using a forward‐viewing echoendoscope in a patient with altered anatomy

**DOI:** 10.1002/deo2.114

**Published:** 2022-04-01

**Authors:** Hiroshi Shimizu, Rei Suzuki, Yuki Sato, Tadayuki Takagi, Naoto Abe, Hiroki Irie, Mitsuru Sugimoto, Takumi Yanagita, Ryoichiro Kobashi, Minami Hashimoto, Tsunetaka Kato, Mika Takasumi, Jun Nakamura, Takuto Hikichi, Hiromasa Ohira

**Affiliations:** ^1^ Department of Gastroenterology Fukushima Medical University Fukushima Japan; ^2^ Department of Endoscopy Fukushima Medical University Hospital Fukushima Japan

**Keywords:** endoscopic retrograde pancreatography, endoscopic ultrasound, forward‐viewing echoendoscope, pancreatic drainage, pancreatic jejunostomy stricture

## Abstract

Pancreatic jejunostomy stricture (PJS) is one of the major late complications after pancreaticoduodenectomy. Endoscopic ultrasound‐guided pancreatic drainage (EUS‐PD) is considered a salvage treatment for symptomatic PJS after endoscopic retrograde pancreatography failure; however, the technical success rate of the endoscopic treatment of PJS remains unsatisfactory, mainly due to surgically altered anatomy. Herein, we describe a case of PJS successfully treated with transjejunal EUS‐PD using a forward‐viewing echoendoscope. A 62‐year‐old man who suffered from repetitive severe back pain due to PJS was referred to our hospital. Since transgastric EUS‐PD was difficult, we attempted transjejunal EUS‐PD using a forward‐viewing echoendoscope. To facilitate scope insertion, we first straightened the afferent jejunal loop and placed a stiff guidewire. With this scheme, we successfully performed transjejunal EUS‐PD and placed a 5‐Fr plastic stent. In conclusion, this technique is useful for treating patients with PJS when transgastric EUS‐PD is difficult.

## INTRODUCTION

Pancreatic jejunostomy stricture (PJS) is one of the major late complications after pancreaticoduodenectomy, accounting for 2%–10%.^1^, ^2^, ^3^ While endoscopic treatments, endoscopic retrograde pancreatography (ERP), or endoscopic ultrasound‐guided pancreatic drainage (EUS‐PD), are the first choice for symptomatic PJS after PD, the technical success rate of the endoscopic treatment of PJS remains unsatisfactory, mainly due to postsurgical altered anatomy.^4^, ^5^, ^6^, ^7^, ^8^ Herein, we describe a case of PJS successfully treated with transjejunal EUS‐PD using a forward‐viewing echoendoscope.

## CASE REPORT

**FIGURE 1:**
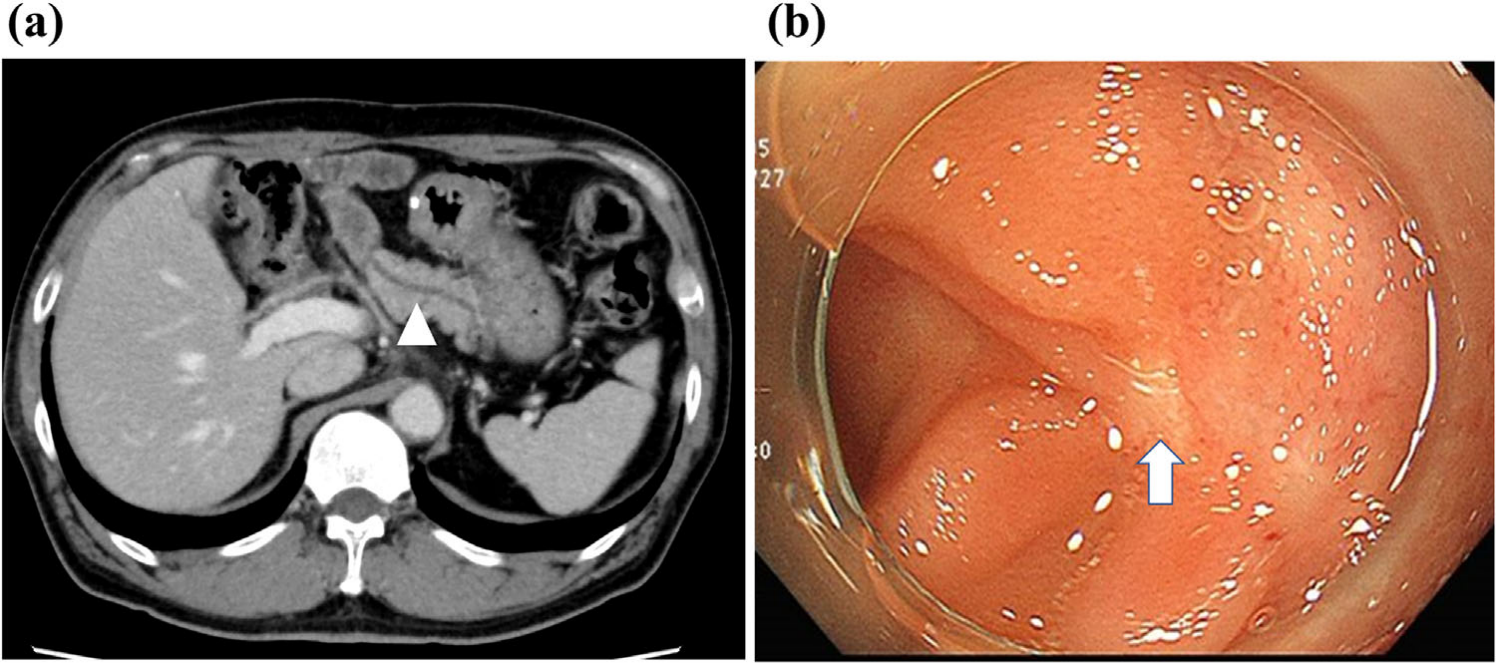
Pretreatment imaging findings. (a) Computed tomography revealed a dilated main pancreatic duct from the anastomosis (arrowhead) in the remnant pancreas. (b) Endoscopic imaging of obstructed pancreatic jejunostomy anastomosis

**FIGURE 2:**
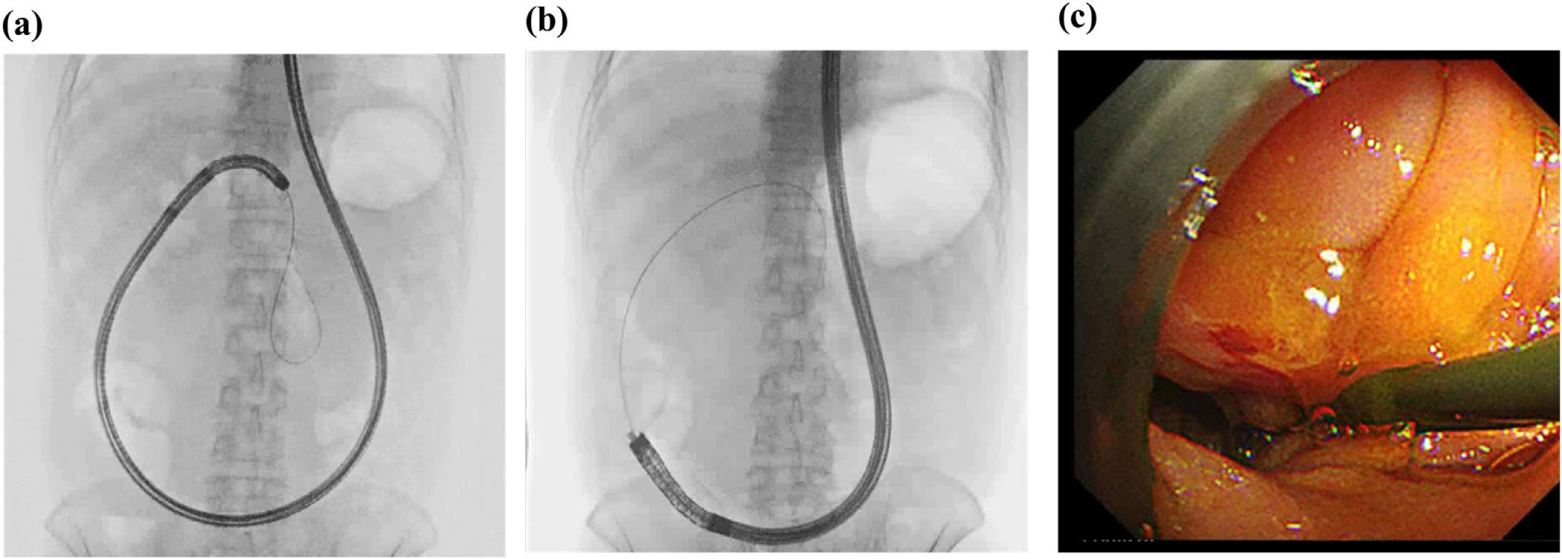
Endoscopic scheme of a forward‐viewing echoendoscope insertion. (a) An enteroscope (SIF‐H290S; Olympus, Tokyo, Japan) was inserted into the end of the afferent jejunal loop. Subsequently, a stiff guidewire was placed. (b,c) A forward‐viewing echoendoscope was inserted along with the guidewire

**FIGURE 3:**
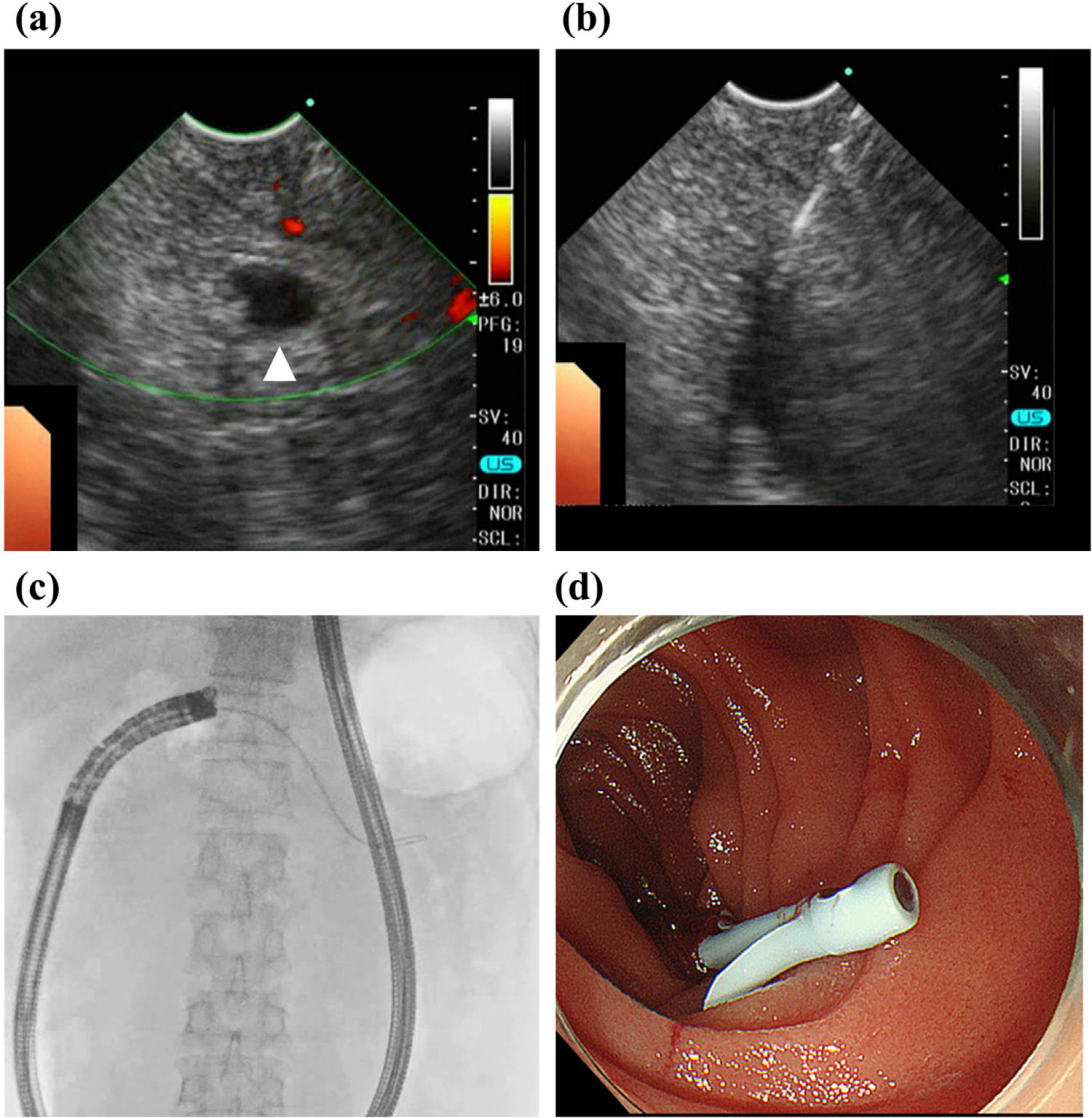
Transjejunal endoscopic ultrasound‐guided pancreatic drainage. (a) Dilated pancreatic duct (5 mm) was detected under anastomosis by endoscopic ultrasound (arrowhead). (b) The pancreatic duct was punctured by a 19‐gauge needle (EZ shot 3 plus; Olympus, Tokyo, Japan). (c,d) A 5‐Fr plastic stent was finally placed in the pancreatic duct

A 62‐year‐old man who had undergone subtotal pancreaticoduodenectomy reconstructed by the modified Child's method with Braun anastomosis for intraductal papillary mucinous adenoma 4 years prior was referred to our hospital for endoscopic treatment for PJS. The man had visited a referral hospital complaining of recurrent severe back pain, and imaging studies at the hospital revealed pancreatic duct dilatation without any evidence of pancreatitis (Figure 1a). While laboratory data did not show any abnormalities, including pancreatic enzymes, there were no other causes of symptoms other than pancreatic dilatation. ERP with an enteroscope (SIF‐H290S; Olympus, Tokyo, Japan) was attempted but failed because the pancreatic jejunostomy was obstructed as a scar (Figure 1b). We firstly planned EUS‐PD from the remnant stomach with an oblique‐viewing linear EUS as the first‐line treatment; however, EUS from the remnant stomach barely revealed pancreatic duct dilatation at the proximal part of the anastomosis. It was difficult to puncture the pancreatic duct from the remnant stomach and insert an oblique‐viewing linear echoendoscope into the deep part of the afferent jejunal loop due to its anatomical condition (e.g., the distance between stomach and pancreatic duct and the maneuverability of echoendoscope). To overcome these limitations and accomplish successful pancreatic drainage, we attempted transjejunal EUS‐PD using a forward‐viewing echoendoscope. We first inserted an enteroscope (SIF‐H290S; Olympus) into the end of the afferent loop and placed a stiff guidewire (1.32 mm in diameter, 4500 mm long; Create Medic, Yokohama, Japan) to facilitate echoendoscope insertion (Figure 2a). After withdrawing the enteroscope, a forward‐viewing echoendoscope (TGF‐UC260J; Olympus) was inserted along the guidewire that was passed through the scope's working channel (Figure 2b&c). The dilated pancreatic duct was successfully visualized immediately beneath the scarred pancreatic jejunostomy anastomosis and punctured using a 19‐gauge needle (EZ shot 3Plus; Olympus) (Figure 3a,b). We inserted a 0.025‐inch guidewire (Visiglide2; Olympus) and then an ERCP catheter (MTW ERCP catheter; Düsseldorf, Germany) along with the guidewire. ERP imaging confirmed successful accession into the pancreatic duct. Furthermore, the puncture site was dilated with a bougie dilator (ES dilator soft type; Zeon Medical Inc., Tokyo, Japan), and a 5‐Fr 9 cm plastic stent (HarmoRay; Hanako Medical Co. Ltd, Saitama, Japan) was placed. Even though the patient was compromised with acute pancreatitis after the procedure, only conservative treatment was required until he totally recovered 5 days after the procedure. Five months after the intervention, the patient is totally asymptomatic, and stent replacement is scheduled.

## DISCUSSION

Symptomatic PJS is one of the most problematic adverse events after pancreaticobiliary surgery. EUS‐PD is considered a salvage technique after ERP failure, but it is sometimes difficult to perform due to anatomical reasons. In the present case, we found that conventional EUS‐PD from the stomach was difficult to perform for the same reasons. While transjejunal EUS‐PD around the PJ anastomosis was an ideal choice, in this case, deep insertion of a conventional lateral‐viewing echoendoscope into the deep part of the afferent loop was expected to be difficult. To overcome this limitation, we utilized a forward‐viewing echoendoscope with the guidance of a stiff guidewire and successfully performed transjejunal EUS‐PD. To the best of our knowledge, this is the first case to use this technique.

The selection of puncture sites is very important in EUS‐PD.^7^ Given the technical difficulty in guidewire manipulation and device delivery, the puncture site should be carefully selected based on the distance between the stomach and pancreatic duct and the size of the pancreatic duct. In cases with surgically altered anatomy with gastrectomy, the selection of a suitable puncture site may be difficult due to the reduced volume of the remnant stomach. If the pancreatic duct is punctured in the region where the stomach and pancreas are far apart, subsequent device delivery can be difficult and may result in treatment failure. Therefore, transjejunal EUS‐PD may be an ideal technique for PJS since the jejunum and pancreatic duct are closely anastomosed.

Insertion of EUS into the deep part of the afferent jejunal loop is a task to be solved for transjejunal EUS‐PD. Two Japanese groups originally reported the usefulness of a forward‐viewing echoendoscope for EUS‐PD in patients with surgically altered anatomy.^9^, ^10^ More recently, Iwai et al. reported a case series that described the usefulness of EUS‐guided drainage using a forward‑viewing echoendoscope for biliopancreatic anastomosis stricture in patients with surgically altered anatomy.^11^ In this report, they performed successful EUS‐guided drainage in eight patients who had undergone Child's reconstruction with Braun anastomosis which was similar to the present case. While they concluded that insertion of a forward‐viewing echoendoscope is feasible and safe, this technique can be difficult if the afferent jejunal loop is long and twisted. Therefore, we utilized a stiff guidewire, which is used in endoscopic long intestinal tube insertion, to modify this technique. After straightening the twisted afferent jejunal loop by an enteroscope, the guidewire was placed to retain the shape of the afferent jejunal loop. Furthermore, by deploying the guidewire into the scope's working channel of a forward‐viewing echoendoscope (ropeway method), scope insertion was performed smoothly.

However, we would like to emphasize the potential risks and difficulties of the presented intervention for patients in a similar situation. First, the stiff guidewire may cause mucosal injury owing to its stiffness. Second, it may be troublesome to replace a pancreatic stent which locates in the deep part of the afferent jejunal loop compared to transgastric EUS‐PD. Third, it can be difficult to insert a forward‐viewing echoendoscope when the afferent loop is long and twisted even with the assistance of a stiff guidewire. Considering these risks and difficulties, the presented intervention can be a salvage treatment when ERCP and transgastric EUS‐PD is difficult to perform or failed.

In conclusion, we recommend transjejunal EUS‐PD for PJS using a forward‐viewing echoendoscope with the assistance of a stiff guidewire in a patient with altered anatomy. We believe this scheme can secure EUS‐PD in patients with surgically altered anatomy.

## CONFLICT OF INTEREST

The authors declare no conflict of interest.

## FUNDING INFORMATION

This research did not receive any specific grant from funding agencies in the public, commercial, or not‐for‐profit sectors.

## ETHICS STATEMENT

Statement on Helsinki Declaration applied for Ethical Committee approval.

## References

[1] Demirjian AN , Kent TS , Callery MP , Vollmer CM . The inconsistent nature of symptomatic pancreatico‐jejunostomy anastomotic strictures. HPB 2010; 12: 482–7. 20815857 10.1111/j.1477-2574.2010.00214.x PMC3030757

[2] Morgan KA , Fontenot BB , Harvey NR , Adams DB . Revision of anastomotic stenosis after pancreatic head resection for chronic pancreatitis: Is it futile? HPB 2010; 12: 211–6. 20590889 10.1111/j.1477-2574.2009.00154.x PMC2889274

[3] Reid‐Lombardo KM , Ramos‐De la Medina A , Thomsen K , Harmsen WS , Farnell MB . Long‐term anastomotic complications after pancreaticoduodenectomy for benign diseases. J Gastrointest Surg 2007; 11: 1704–11. 17929105 10.1007/s11605-007-0369-7

[4] Farrell J , Carr‐Locke D , Garrido T , Ruymann F , Shields S , Saltzman J . Endoscopic retrograde cholangiopancreatography after pancreaticoduodenectomy for benign and malignant disease: Indications and technical outcomes. Endoscopy 2006; 38: 1246–9. 17163327 10.1055/s-2006-944970

[5] Chahal P , Baron TH , Topazian MD , Petersen BT , Levy MJ , Gostout CJ . Endoscopic retrograde cholangiopancreatography in post‐Whipple patients. Endoscopy 2006; 38: 1241–5. 17163326 10.1055/s-2006-945003

[6] Kinney TP , Li R , Gupta K , *et al*. Therapeutic pancreatic endoscopy after Whipple resection requires rendezvous access. Endoscopy 2009; 41: 898–901. 19750454 10.1055/s-0029-1215081

[7] Nakai Y , Kogure H , Isayama H , Koike K . Endoscopic ultrasound‐guided pancreatic duct drainage. Saudi J Gastroenterol 2019; 25: 210–7. 30632484 10.4103/sjg.SJG_474_18 PMC6714474

[8] Giovannini M . Endoscopic ultrasound‐guided pancreatic duct drainage: Ready for the prime time? Endosc Ultrasound 2017; 6: 281–4. 29063870 10.4103/eus.eus_86_17 PMC5664847

[9] Hodo Y , Shirota Y , Suda T , Wakabayashi T . Successful EUS‐guided retrograde pancreatic duct stent placement for refractory pancreaticojejunostomy stricture after pancreaticoduodenectomy with a forward‐viewing echoendoscope. VideoGIE 2018; 3: 196–8. 30128386 10.1016/j.vgie.2018.03.009 PMC6098672

[10] Nakaji S , Hirata N , Shiratori T , Kobayashi M , Yamauchi K . Endoscopic ultrasound‐guided pancreaticojejunostomy with a forward‐viewing echoendoscope as a treatment for stenotic pancreaticojejunal anastomosis. Endoscopy 2015; 47: E41–2. 25603522 10.1055/s-0034-1391245

[11] Iwai T , Kida M , Yamauchi H , *et al*. EUS‐guided transanastomotic drainage for severe biliopancreatic anastomotic stricture using a forward‐viewing echoendoscope in patients with surgically altered anatomy. Endosc Ultrasound 2021; 10: 33–8. 33473043 10.4103/eus.eus_72_20 PMC7980695

